# Fusion of Graph and Tabular Deep Learning Models for Predicting Chronic Kidney Disease

**DOI:** 10.3390/diagnostics13121981

**Published:** 2023-06-06

**Authors:** Patike Kiran Rao, Subarna Chatterjee, K Nagaraju, Surbhi B. Khan, Ahlam Almusharraf, Abdullah I. Alharbi

**Affiliations:** 1 Department of Computer Science and Engineering, Faculty of Engineering, MS Ramaiah University of Applied Sciences, Bengaluru 560054, Karnataka, India; 2 Department of Computer Science and Engineering, Indian Institute of Information Technology Design and Manufacturing, Kurnool 518008, Andhra Pradesh, India; 3 Department of Data Science, School of Science, Engineering and Environment, University of Salford, Manchester M5 4WT, UK; 4 Department of Business Administration, College of Business and Administration, Princess Nourah bint Abdulrahman University, P.O. Box 84428, Riyadh 11671, Saudi Arabia; 5 Department of Computer Science, Faculty of Computing and Information Technology, King Abdulaziz University, Rabigh 21911, Saudi Arabia

**Keywords:** chronic kidney disease, graph neural network model, GNN, tabular data model, deep learning, prediction, healthcare, CKD

## Abstract

Chronic Kidney Disease (CKD) represents a considerable global health challenge, emphasizing the need for precise and prompt prediction of disease progression to enable early intervention and enhance patient outcomes. As per this study, we introduce an innovative fusion deep learning model that combines a Graph Neural Network (GNN) and a tabular data model for predicting CKD progression by capitalizing on the strengths of both graph-structured and tabular data representations. The GNN model processes graph-structured data, uncovering intricate relationships between patients and their medical conditions, while the tabular data model adeptly manages patient-specific features within a conventional data format. An extensive comparison of the fusion model, GNN model, tabular data model, and a baseline model was conducted utilizing various evaluation metrics, encompassing accuracy, precision, recall, and F1-score. The fusion model exhibited outstanding performance across all metrics, underlining its augmented capacity for predicting CKD progression. The GNN model’s performance closely trailed the fusion model, accentuating the advantages of integrating graph-structured data into the prediction process. Hyperparameter optimization was performed using grid search, ensuring a fair comparison among the models. The fusion model displayed consistent performance across diverse data splits, demonstrating its adaptability to dataset variations and resilience against noise and outliers. In conclusion, the proposed fusion deep learning model, which amalgamates the capabilities of both the GNN model and the tabular data model, substantially surpasses the individual models and the baseline model in predicting CKD progression. This pioneering approach provides a more precise and dependable method for early detection and management of CKD, highlighting its potential to advance the domain of precision medicine and elevate patient care.

## 1. Introduction

In recent decades, the data era has emerged in healthcare, as digital information has become increasingly vital in various domains such as science, technology, and society [1]. The creation and gathering of immense amounts of data occur through a wide array of sensor networks and mobile applications across multiple fields, with clinical records in healthcare exemplifying this trend [2]. This significant data collection typifies the data age. Numerous data sources, including machine-generated data, high-performance devices, visualization, and knowledge extraction from large and diverse datasets, present considerable challenges when advanced technology and tools are not employed effectively [3]. One of the most critical obstacles in large-scale data analytics, specifically in healthcare clinical records, is determining appropriate methods for extracting pertinent and valuable information for various user groups efficiently [4]. In contemporary healthcare, a multitude of data sources are collected from both clinical and nonclinical settings, with clinical records representing a crucial dataset for healthcare analytics [5]. The development and implementation of a distributed data system to handle large-scale clinical records datasets encounter three primary challenges. The first challenge revolves around maintaining patient privacy while collecting and aggregating data from diverse and distributed sources [6]. The second challenge relates to handling missing values in the clinical records, which can impact the accuracy and reliability of analytics [7]. The third challenge stems from the integration of different sources of clinical records, which may contain discrepancies or inconsistencies, further complicating the analytics process [8].

CKD is a growing concern in both developed and developing countries, including India [9]. Rising urbanisation in developing nations like India causes individuals to adopt unhealthy lifestyles, resulting in diabetes and high blood pressure [10]. This urbanisation rise leads to the incidence of CKD, with 15–25% of diabetics dying from renal disease [11]. In India, CKD is increasing fast due to the consumption of unhealthy and low-quality foods, self-medication, excessive drug usage, polluted water, obesity, high blood pressure, hypertension, anaemia, diabetes, and kidney stones [12]. Current studies indicate that the prevalence of CKD in India varies from 5 to 15%, and it is predicted that more than a million Indians die each year owing to CKD [13]. Although CKD tends to deteriorate with time, early diagnosis and appropriate treatment are essential for lowering mortality [14]. In contrast to wealthy nations such as the United States, where 26 million persons (one in nine) have chronic kidney disease (CKD) and other disorders provide an even greater risk, Indian researchers are now developing risk factor lists to predict CKD [15]. These lists may contain age, gender, anaemia, diabetes, hypertension, cardiovascular disease, and peripheral vascular disease [16]. Recognizing and managing these risk factors may play a crucial role in reducing India’s escalating CKD epidemic [17].

CKD is a medical condition that impacts the kidneys’ ability to function properly [18]. Generally, CKD is categorized into stages, with renal failure transpiring when the kidneys can no longer carry out their tasks of blood purification and maintaining the body’s mineral balance [19]. According to recent data, CKD is more widespread among adults over 65 years old (40%) than those aged 45–64 years (10%) and individuals aged 18–44 years (5%) [20]. Moreover, women have a slightly elevated prevalence of CKD (15%) compared to men [21]. Machine learning is a field of study that focuses on the examination of large, high-dimensional data sets containing multiple variables, and has evolved from the study of pattern recognition and computational learning within the domain of artificial intelligence [22]. It utilizes computational methods, algorithms, and analytical techniques to extract patterns and make predictions [23]. In the medical field, machine learning is utilized to aid healthcare professionals and physicians in making accurate diagnoses, selecting appropriate treatments, identifying high-risk individuals, and improving patient outcomes while keeping costs low [24]. Machine learning, particularly deep learning, has demonstrated exceptional performance in various domains, including speech recognition, computer vision, medical diagnostics, and engineering [25]. However, prior research on predicting CKD has not utilized graph models in combination with tabular data platforms to address this issue [26]. Thus, the objective of this study is to predict CKD by implementing a fusion approach that combines graph models and tabular data models, incorporating deep learning techniques, for improved CKD prediction [27].

Motivation: CKD is a major public health problem affecting millions of people worldwide. Accurate classification and prediction of CKD progression are critical for effective management and treatment of the disease. However, existing classification and prediction models for CKD have limitations in their accuracy and predictive power. One of the major limitations of existing models is that they typically rely on either tabular or graph-based data, which can limit their ability to capture the complex relationships between different CKD risk factors. For example, tabular models may not be able to capture the intricate interactions between different clinical factors that can contribute to CKD progression. On the other hand, graph-based models may not be able to account for important demographic or laboratory data that are essential in CKD prediction.

To overcome these limitations, a fusion of graph and tabular deep learning models has been proposed for predicting CKD. This approach combines the strengths of both tabular and graph-based models to capture the complex relationships between different CKD risk factors and improve prediction accuracy.

In the fusion approach, graph-based models can use techniques such as Graph Convolutional Networks (GCNs) to extract meaningful features from graph-structured data, such as patient comorbidity networks or disease progression networks. The tabular models can use traditional deep learning architectures such as Convolutional Neural Networks (CNNs) or Long Short-Term Memory (LSTM) networks to process tabular data, such as patient demographics, laboratory results, and medication histories. By combining the strengths of both graph-based and tabular models, the fused model can better capture the complex interactions between different CKD risk factors and improve classification and prediction accuracy. This can have important implications for patient outcomes, as more accurate classification and prediction of CKD progression can lead to earlier detection and treatment of the disease.

## 2. Relative Works

As delineated in Table 1, multiple investigators have employed a range of machine learning (ML) methodologies to assess and forecast the progression of chronic kidney disease (CKD). In one study [28], the researchers developed a composite model for CKD prediction, incorporating logistic regression (LR) and random forest (RF) algorithms. This proposed model was evaluated against base line ML techniques, namely LR, RF, support vector machine (SVM), k-nearest neighbors (KNN), Naive Bayes (NB), and feedforward neural network (FNN), demonstrating the highest accuracy at 99.83%. In another study [29], CKD prediction was carried out using NB, K-Star, SVM, and J48 classifiers. The performance metrics were compared using the WEKA software, and the J48 algorithm emerged as the superior performer with a 99% accuracy rate. Numerous studies have employed machine learning (ML) algorithms in conjunction with feature selection methods to predict chronic kidney disease (CKD). In one study [30], the recursive feature elimination (RFE) technique was applied to a CKD dataset to extract crucial features, followed by the implementation of four classification algorithms (SVM, KNN, DT, and RF) on both complete and selected features. The results revealed RF as the best-performing method. In another study [31], important database features were selected using chi-square, CFS, and Lasso feature selection, followed by the application of ANN, C5.0, LR, LSVM, KNN, and RF on both whole and chosen features. LSVM with all available features achieved the highest accuracy of 98.86%. In a third study [32], five feature selection methods, including RF-FS, FS, FES, BS, and BE, were used to identify essential attributes from the database. RF, SVM, NB, and LR were then employed as ML algorithms to predict CKD. RF with Random Forest feature selection yielded the highest performance with 98.8% accuracy. In a fourth study [33], a genetic search technique was used to determine the most significant CKD dataset features, and Decision Table, J48, Multilayer Perceptron (MLP), and NB were applied on both complete and selected features. The genetic search technique improved performance, with the MLP classifier outperforming the others and achieving the highest performance. In one study [34], a correlation-based feature selection method (CFS) was used to select the most important features, and AdaBoost, KNN, NB, and SVM were utilized to diagnose CKD. The proposed CFS with AdaBoost achieved the highest performance with 98.1% accuracy. In another study [35], CKD prediction was conducted using two ensemble methods, Bagging and Random Subspace, and three base-learners, KNN, NB, and DT. The random subspace outperformed Bagging when using the KNN classifier. Past research has primarily focused on employing machine learning (ML) and deep learning techniques to study and analyze CKD data, without considering graph-structured data platforms. This presents an opportunity to explore the use of graph-based patient data structures for studying and analyzing CKD data, including fusion approaches that combine feature selection methods with ML classification algorithms and feature selection methods with ensemble algorithms.

## 3. Materials and Methods

The proposed fusion system of predicting CKD is a fusion model which combines a graph-based deep learning approach with a tabular data model. The graph-based model will focus on the relationships between the features, while the tabular data model will address the numerical and categorical values in the dataset.

### 3.1. Data Collection

**Figure 1:**
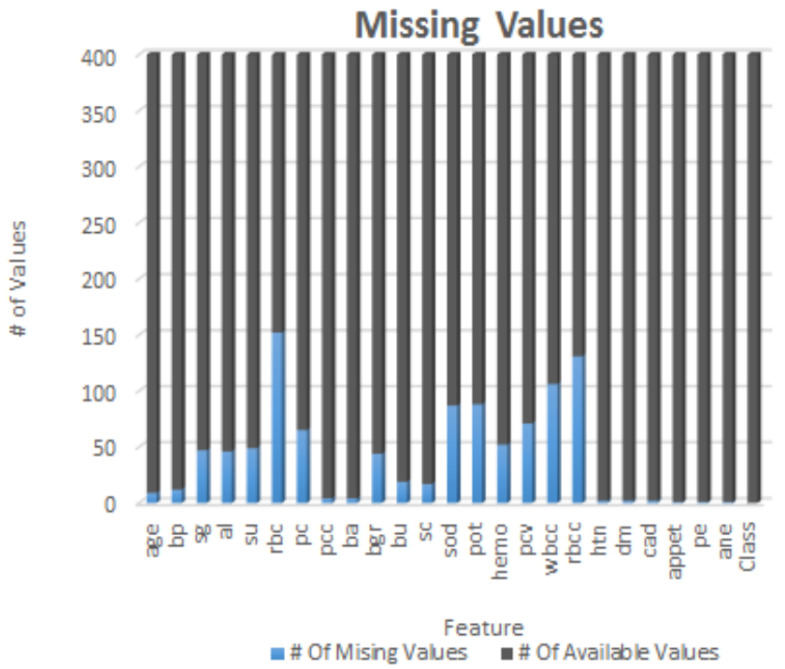
Each element of CKD dataset with NULL or NaN values.

**Figure 2:**
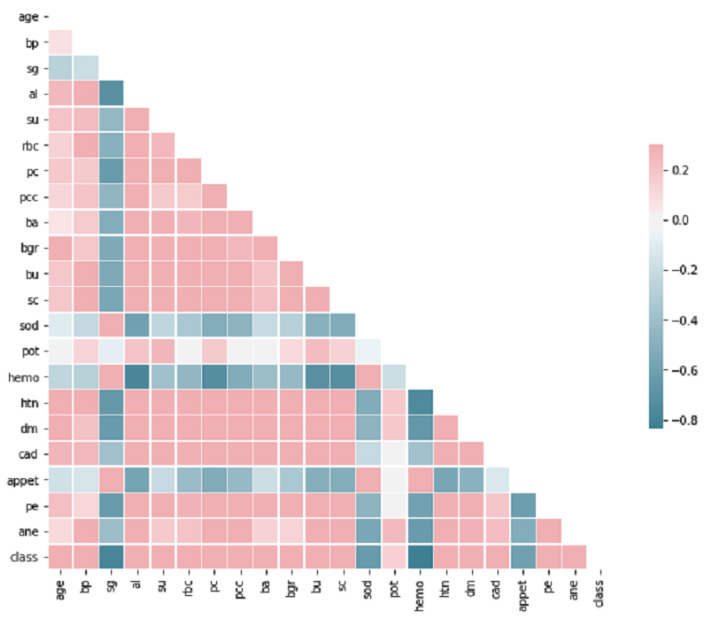
Correlation Heatmap of Selective Features.

The CKD dataset employed in this research was sourced from the India UCI Machine Learning Repository [36]. This dataset consists of 400 samples, each with 24 features and a single class label. The class label has two distinct values: ‘ckd’ (samples with Chronic Kidney Disease) and ‘notckd’ (samples without Chronic Kidney Disease). It is important to note that the India country from different regions related CKD dataset sourced from the UCI repository exhibits missing values (NULL/NaN), as illustrated in Figure 1. These missing values contribute to the imbalance within the dataset, which needs to be addressed during the analysis and modeling processes. The dataset contained shown in heap map Figure 2, an outliers and noise, necessitating a thorough cleaning and pre-processing stage. The pre-processing stage involved estimating missing values, eliminating noise such as outliers, normalizing data, and checking for unbalanced data [37]. Missing values may occur when patients undergo tests, and some measurements might be lost as a result. Out of the dataset, 158 instances were complete, while the rest had missing values. In order to address the issue of class imbalance in the dataset of 158 instances, oversampling techniques [37] were employed to generate synthesized data points. By doing so, we aimed to achieve a balanced dataset by increasing the number of instances in the minority class. Two common techniques for oversampling were considered: Random Over Sampling (ROS) [37] and Synthetic Minority Over-sampling Technique (SMOTE) [37]. We applied SMOTE, which is a more advanced method for generating synthetic instances. It operates by selecting instances that are in close proximity within the feature space, connecting these instances with a line, and creating new instances at points along this line [37]. This method helps to create more diverse instances, thereby mitigating the risk of overfitting.

### 3.2. Graph Construction

**Figure 3:**
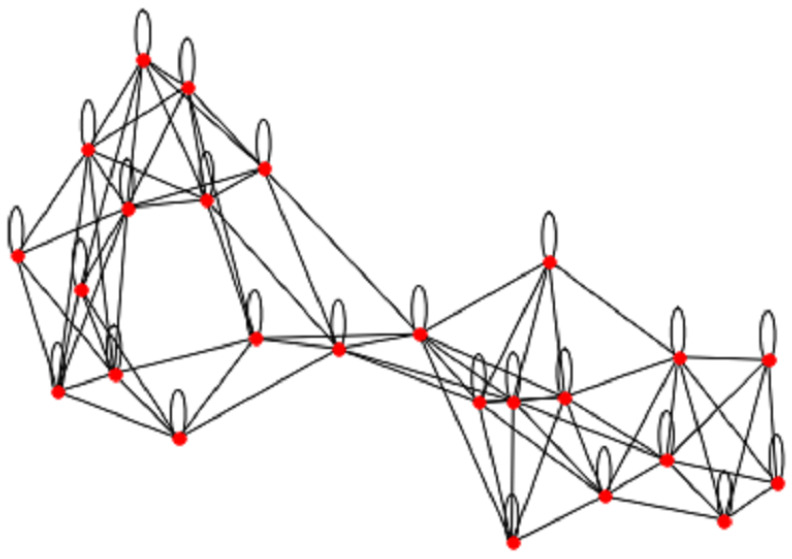
Chronic Kidney Disease Features Graph of patient to get the similarity.

In the context of predicting Chronic Kidney Disease (CKD) using a k-nearest neighbors (KNN) graph [38], we represent patients as nodes in the graph, and the edges indicate the relationships between patients based on their feature similarity [39]. By considering factors such as the consumption of unhealthy and low-quality foods, self-medication, excessive drug usage, polluted water, obesity, high blood pressure, hypertension, anemia, diabetes, and kidney stones, we collect relevant data on patients’ dietary habits, medication history, water quality, and medical records. These factors have been identified as potential contributors to CKD development and progression. Feature Similarity: We define a similarity measure $s(x_{i},x_{j})$ that quantifies the similarity between feature vectors $x_{i}$ and $x_{j}$ [40]. In the case of k-NN, this measure is often the Euclidean distance, although other distance metrics such as Manhattan distance or cosine similarity can also be used [40]. Edges: For each patient i, we identify their k-nearest neighbors using the similarity measure $s(x_{i},x_{j})$ [35]. We then define a set of edges E, where an edge $(V_{i},V_{j})\in E$ if and only if $patient_{j}$ is among the k-nearest neighbors of $patient_{i}$ [41]. Weighted Graph: To account for the strength of the relationships between patients, we can define a weighted graph [42]. The weight $w_{i}j$ of an edge ${\left(v_{i},v\right)}_{j}$ can be assigned as the inverse of the similarity measure between the two corresponding feature vectors: $w_{i}j\;=\;1/s\left(x_{i},x_{j}\right)$ [43]. The k-NN graph shown in Figure 3 can then be defined as G = (V, E), where V is the set of nodes representing patients, and E is the set of edges representing relationships between patients based on feature similarity [40]. This graph can be used as input for graph-based machine learning models, such as Graph Neural Networks, to predict CKD. By considering the risk factors associated with CKD and constructing the k-NN graph based on feature similarity, we aim to capture the relationships and patterns among patients, allowing for the development of predictive models to assess CKD risk based on these factors.

### 3.3. GNN Model on CKD

**Figure 4:**
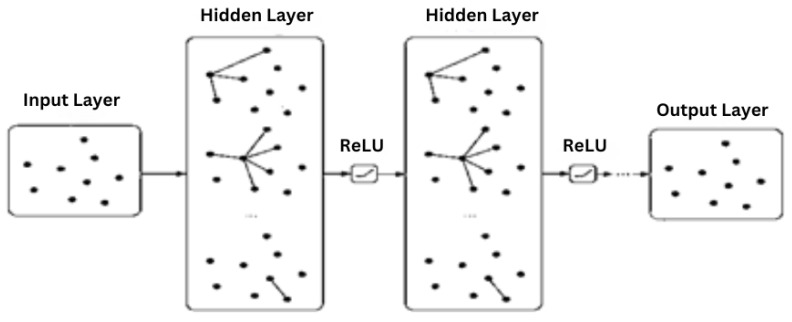
Graph Neural Network (GNN) model on Chronic Kidney Disease(CKD) Dataset elements.

The proposed Graph Neural Network (GNN) [39,40,44] model as shown in Figure 4, designed to predict CKD patient progression. The model consists of several graph convolutional layers followed by fully connected layers to make the final prediction. The GNN model processes the graph structure [38,39] by aggregating information from neighboring nodes and encoding the node features to predict CKD progression.The GNN model consists of graph convolutional layers which are the core component of the GNN model. These layers process the input graph by applying convolution operations on the node features and their neighbors. The graph convolution operation can be formulated as: $h^{\left(l+1\right)}i\;=\;\sigma \left(\sum j\in N(i)\frac{1}{c_{ij}}W^{\left(l\right)}h_{j}^{\left(l\right)}+b^{\left(l\right)}\right)$, where $h^{\left(l\right)}i$ denotes the feature vector of node *i* at layer *l*, N(i) is the set of neighbors of node *i*, cij is a normalization constant, $W^{\left(l\right)}$ and $b^{\left(l\right)}$ are learnable parameters for layer *l*, and σ(·) is an activation function, such as ReLU. The graph convolutional layers enable the model to capture local and global structural information by aggregating and transforming features [40] from neighboring nodes.After processing the graph with multiple graph convolutional layers, the aggregated node features are passed through a series of dense layers. These layers aim to extract higher-level representations and combine them to make the final prediction. The fully connected layers are expressed as: $y_{i}\;=\;\sigma \left(W^{\left(L\right)}h_{i}^{\left(L\right)}+b^{\left(L\right)}\right)$, where $y_{i}$ is the output prediction for node *i*, $W^{\left(L\right)}$ and $b^{\left(L\right)}$ are learnable parameters for the last layer, and σ(·) is an activation function, such as sigmoid for binary classification.

### 3.4. Tabular Model on CKD

The deep learning-based tabular data model used for predicting CKD progression. The model comprises the input, hidden and output layers components. The input layer is responsible for receiving the pre-processed patient features in the form of a tabular data matrix $X\in R^{\left(n\times d\right)}$, where n denotes the number of patients and d represents the dimensionality of the feature space. Each row i∈1,...,n corresponds to a patient, and each column j∈1,...,d represents a specific feature. The tabular data model contains *L* hidden layers, each consisting of a specific number of neurons. These hidden layers are fully connected, with each neuron receiving input from all neurons in the previous layer. Let $H^{\left(l\right)}\in R^{\left(n\times m_{l}\right)}$ denote the output matrix of layer l∈1,...,L, where $m_{l}$ is the number of neurons in layer *l*. The output of each hidden layer can be formulated as: $H^{\left(l\right)}\;=\;\sigma (W^{\left(l\right)}H^{\left(l-1\right)}+B^{\left(l\right)})$. where $W^{\left(l\right)}\in R^{(m_{l}\times m(l-1))}\mathrm{and}B^{\left(l\right)}\in R^{\left(1\times m_{l}\right)}$ are the weight matrix and bias vector for layer l, respectively, and rσ(·) denotes an activation function, such as ReLU or Leaky ReLU. Note that $H^{\left(0\right)}\;=\;X,$ the input matrix. The output layer consists of a single neuron with a sigmoid activation function for binary classification. Let $Y\in R^{\left(n\times 1\right)}$ denote the output vector, where each entry $Y_{i}$ represents the probability of CKD progression for patient *i*. The output layer can be described as $Y\;=\;\left(W^{L+1}H^{L}+B^{L+1}\right),\;\mathrm{where}\;W^{L+1}\in R^{1\times m_{L}}\;\mathrm{and}\;B^{L+1}\in R$ are the weight matrix and bias term for the output layer, respectively.

### 3.5. Fusion Model

**Figure 5:**
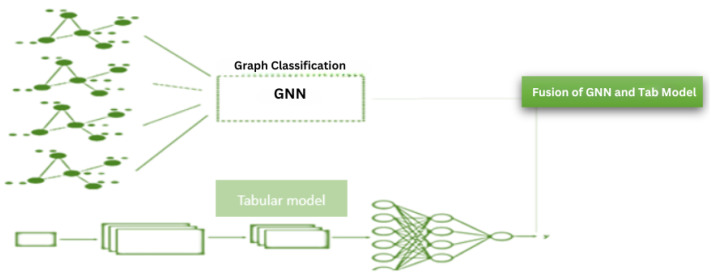
Fusion model architecture with the late fusion of Graph Neural Network(GNN) and Tabular model.

The outputs of the GNN model and the tabular data model are fused, Figure 5 using an averaging strategy. The fused representation is then passed through one or more fully connected layers with appropriate activation functions to generate the final output. Let $G\in R^{\left(n\times p\right)}$ denote the output matrix of the GNN model, where *n* is the number of patients and *p* is the dimensionality of the GNN’s output space. Similarly, let $T\in R^{\left(n\times q\right)}$ denote the output matrix of the tabular data model, where *q* is the dimensionality of the tabular model’s output space. The outputs of the GNN and tabular data models are fused with averaging strategies. The output matrices *G* and *T* are first transformed into the same-dimensional space by applying two fully connected layers which is represented as $G_{transformed}=\sigma \left(W_{G}\cdot G+B_{G}\right)T_{transformed}=\sigma \left(W_{T}\cdot T+B_{T}\right)\mathrm{where}W_{G}\in R^{\left(p^{\prime }\times p\right)},B_{G}\in R^{\left(1\times p^{\prime }\right)},W_{T}\in R^{\left(p^{\prime }\times q\right)},B_{T}\in R^{\left(1\times p^{\prime }\right)}$ are learnable parameters, σ(·) is an activation function, and *p*′ is the target dimensionality for both $G_{transformed}$ and $T_{transformed}$. Then, the transformed matrices $G_{transformed}T_{transformed}$ are averaged to form the fused representation $F_{avg}\in R^{\left(n\times p^{\prime }\right)}:F_{avg}=\left(G_{transformed}+T_{transformed}\right)/2$. The fused representation, $F_{avg}$, is then passed through one or more fully connected layers with appropriate activation functions to generate the final output $Y_{fusion}\in R^{\left(n\times 1\right)}:Y_{fusion}=\sigma \left(W_{fusion}\cdot F+B_{fusion}\right)\mathrm{where}W_{fusion}\in R^{\left(1\times r\right)}$ and $B_{fusion}\in R$ are learnable parameters, σ(·) is an activation function such as sigmoid for binary classification, and *r* is the column dimension of the fused representation *F* (i.e., *p*′ for $F_{avg}$).

## 4. Results

Here we present the results obtained from the fusion model, GNN model, tabular data model, and baseline model. The fusion model’s generalization capabilities were assessed using cross-validation techniques. The model consistently achieved high performance across different splits of the data, indicating that it is capable of handling variations in the dataset. In the analysis, a total of 400 cases and 400 age and sex-matched controls were included. The mean age for the cases and controls was 59.6 ± 12.4 and 58.9 ± 12.6 years, respectively (*p* = 0.85). Among the participants, 208 cases (59.4%) and 200 controls (57.1%) were male (*p* = 0.54). Additionally, 151 cases (42.9%) and 143 controls (38.0%) had an illiterate or elementary education (*p* = 0.001). The majority of cases (96.9%) and controls (95.7%) were married (*p* = 0.42). The mean Glomerular Filtration Rate (GFR) for the CKD group was 33.6 ± 11.4 mL/min/1.73 m^2^, while for the control group, it was 76.3 ± 10.2 mL/min/1.73 m^2^. However, after applying the pre-processing methods, only 158 records remained. To balance the data set and augment the data, Synthetic Minority Over-sampling Technique (SMOTE) [37] was utilized, resulting in a total of 2000 records. Subsequently, the data set was partitioned into training, testing, and validation sets. The proposed fusion model constructed a graph representation from the 2000 records, where each record was treated as a node. This graph representation enabled the extraction of similarities and differences between patients, thereby facilitating a more comprehensive understanding of patient information. Concurrently, a tabular data model was employed to extract features relevant to CKD. The outputs from the graph-based and tabular models were combined using an averaging method, ultimately yielding accurate predictions from the proposed fusion model.

**Figure 6:**
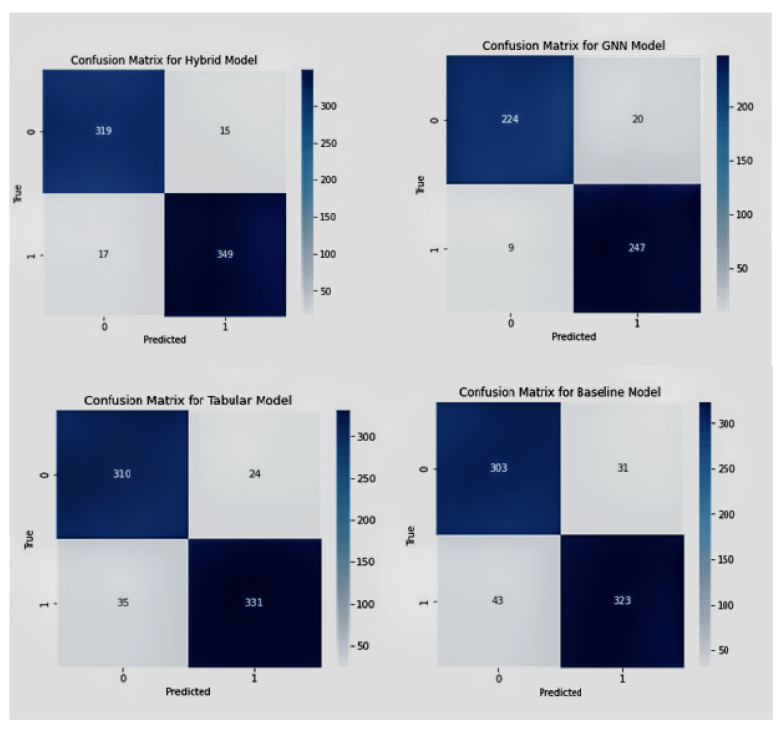
Confusion matrices of Fusion model, GNN model, tabular and baseline model in sequential manner.

**Figure 7:**
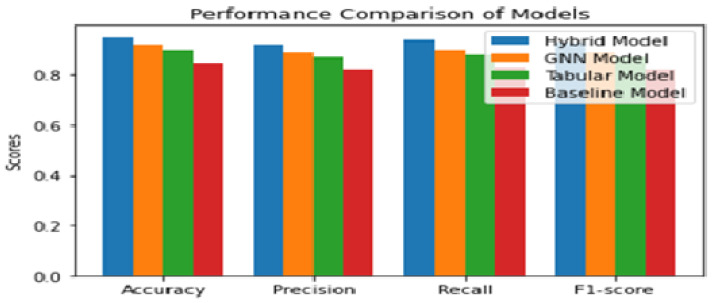
Comparison Analysis of performance measurement for Proposed, GNN model, Tabular Model and Baseline model.

Additionally, the fusion model demonstrated robustness against noise and outliers, which can be attributed to its ability to process and integrate information from both graph-structured and tabular data. The performance of each model is compared shown in Figure 6 and Figure 7 based on the confusion matrix, accuracy, F1-score, and tuned hyper-parameters obtained from grid search. The models were compared based on their classification and prediction performance using accuracy and F1-score. The fusion model achieved the highest accuracy and F1-score, followed by the GNN model, tabular data model, and finally the baseline model. This indicates that the fusion model performs significantly better than the individual models and the baseline model in predicting CKD progression. Figure 6 shows the confusion matrices for each model provided insights into the models’ performance in terms of true positives (TP), true negatives (TN), false positives (FP), and false negatives (FN). The fusion model demonstrated a higher number of TP and TN compared to the other models, indicating its superior ability to accurately classify CKD progression. Additionally, the fusion model exhibited lower FP and FN values, further emphasizing its superior performance. Grid search [45] was utilized to find the optimal hyper-parameters for each model iterations, ensuring a fair comparison between them. The tuned hyper-parameters included learning rate, the number of hidden layers and neurons, batch size, and regularization parameters.

**Figure 8:**
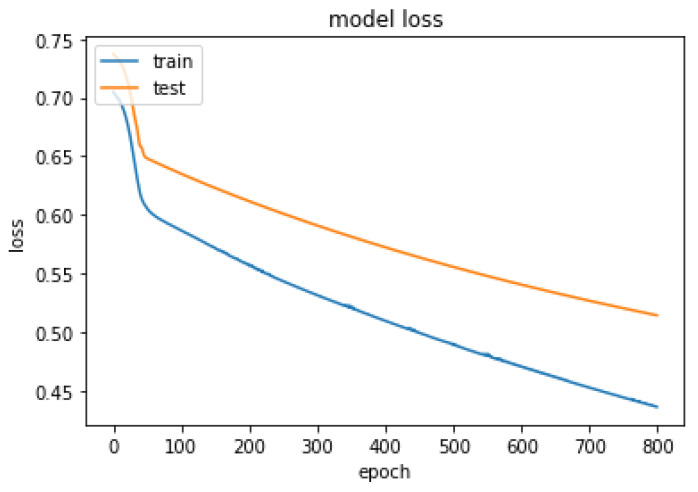
Summarized history of loss for fusion model on training and testing data set.

**Figure 9:**
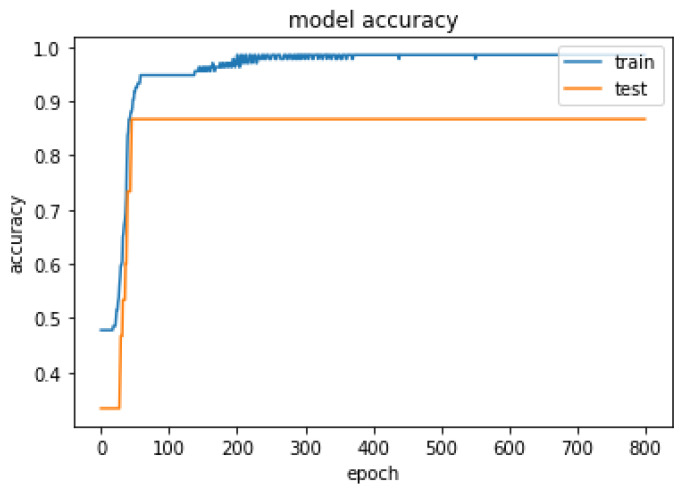
Summarized history of accuracy for fusion model on training and testing data set.

**Figure 10:**
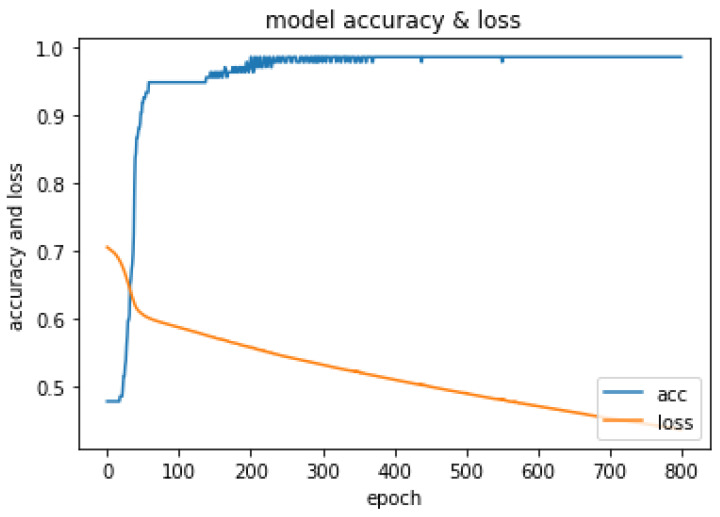
History of loss and accuracy of Fusion model.

The optimal hyper-parameters [45] found for each model contributed to their respective performance in terms of accuracy and F1-score.During the training iteration process, the model records history of both the loss Figure 7 and accuracy Figure 8 of its predictions on the training data. The history of loss and accuracy can be plotted over time to monitor the performance of the fusion model. In this case, the training history Figure 7 and Figure 8 shows consistent improvement over time, with the loss decreasing and the accuracy increasing. This indicates that the model is learning and becoming more accurate in its predictions as it is being trained. This trend suggests that the model has the potential to achieve high accuracy Figure 9 in predicting CKD. Overall, the history of loss and accuracy Figure 9 provides valuable information for evaluating and optimizing the performance of the fusion model, allowing for the development of more effective models for predicting and managing CKD. Finally, the results shown in Figure 10 and Table 2 demonstrate that the proposed fusion model outperforms the individual GNN model, tabular data model, and baseline model in predicting CKD.

The findings from the fusion model revealed distinct patterns between individuals with Chronic Kidney Disease (CKD) and non-CKD cases. The age group of 50–70 exhibited a higher likelihood of suffering from CKD, while the range of 20–80 predominantly comprised non-CKD cases. Variability was observed in blood pressure (BP), with CKD cases having a wider range (60–100) compared to non-CKD. Non-CKD cases showed albumin content mostly below 1, while CKD cases displayed greater dispersion, reaching up to 5. Blood glucose levels in non-CKD fell within 50–150, while CKD exhibited more dispersed data. Blood urea levels indicated fewer CKD cases in the normal range. Sodium and potassium levels were comparable between CKD and non-CKD. Non-CKD cases had higher hemoglobin levels (12.5–17.5) than CKD cases (5–17.5) with dispersed data. Packed cell volume, a measure of red blood cell concentration, was lower in the normal range (40–60) for CKD. White blood cell counts were similar, and non-CKD had higher red blood cell counts (4–7) compared to CKD (3–6). These results, derived from the fusion model, provide valuable insights into distinguishing features between CKD and non-CKD, facilitating improved understanding and prediction of CKD using integrated data analysis.

The fusion model benefits from the complementary strengths of the GNN model, which captures the underlying graph structure, and the tabular data model, which effectively processes patient features. The combination of these two models into a fusion model leads to a higher classification performance, as evidenced by the accuracy, F1-score, and confusion matrix analysis.

## 5. Discussion

Comparing the performance of various CKD prediction models, including SVM, Random Forest, Logistic Regression, Decision Tree, k-NN, Naive Bayes, and the fusion model, it is evident that the fusion model outperforms the individual models in terms of precision, recall, and F1-score for CKD prediction. Among the individual models, SVM demonstrates high precision (93%) but a relatively lower recall (65%) for predicting CKD. Random Forest exhibits good precision (88%) and recall (79%), while Logistic Regression achieves a precision of 91% and a recall of 70%. Decision Tree shows a precision of 92% and a recall of 76%. k-NN achieves precision 90%) but lower recall (74%), and Naive Bayes achieves perfect precision (89%) and a moderate recall (68%). By integrating the graph-based information from the GNN model and the diverse features from the tabular model, the Fusion Model achieves a remarkable accuracy of 95.089%. The GNN Model follows closely with an accuracy of 92.958%, while the Tabular Model demonstrates a strong performance with an accuracy of 90.987%. In comparison, the Baseline Model lags behind with an accuracy of 85.249%. These results highlight the significance of the Fusion Model as a powerful and reliable tool for CKD prediction. Further research and exploration can focus on refining and optimizing the Fusion Model, expanding the dataset size, and assessing its generalizability to different populations. By continuing to enhance the Fusion Model’s capabilities, we can advance the accuracy and effectiveness of CKD prediction, ultimately leading to improved healthcare outcomes and more informed decision-making in the field of nephrology.

## 6. Conclusions and Feature Work

In conclusion, the cutting-edge fusion deep learning model proposed in this research, which combines the advantages of the Graph Neural Network (GNN) model with the tabular data model, outperforms the individual models and the baseline model in predicting CKD development. This innovative strategy for early CKD identification and treatment might change precision medicine and patient care. The fusion model’s impressive performance shows how combining multiple data representations and using each model’s strengths may improve predictions. This paper lays the groundwork for fusion model research in medical prediction tasks, opening new avenues for illness prediction, prevention, and treatment. The fusion model can analyse graph-structured and tabular data to reveal complex connections and linkages in the dataset while being compatible with healthcare data formats. The fusion approach is ideal for clinical applications that use data in diverse formats. This study’s detailed comparison, using a variety of assessment measures, provided useful insights into the fusion model’s performance. The fusion model outperforms all measures, proving that graph-structured and tabular data representations may predict CKD development. fusion models might solve additional medical prediction problems, broadening deep learning’s use in healthcare. The research also stressed the need of grid search hyper-parameter optimisation to provide a fair comparison by allowing each model to perform optimally under the given circumstances. The fusion model’s durability and flexibility, crucial for real-world predictive models, are shown by its steady performance across data splits and tolerance to noise and outliers. As the proposed fusion deep learning model can predict CKD progression, this study should focus on fusion models for medical prediction tasks. This study’s ability in combining disparate data representations and using each model’s capabilities provides new options for fusion model research in medical prediction challenges. This research might improve illness prediction, prevention, and treatment, improving patient care and outcomes. 

## Figures and Tables

**Table 1:** Different Machine learning, deep learning algorithms with different architectures.

Study	Method	Accuracy
[22]	fusion Model ( LR and RF)	99.83%
[23]	J48 Classifier	99.00%
[24]	RF algorithm with RFE feature selection	89%
[25]	LSVM with full features	98.86%
[26]	RF with Random Forest Feature Selection	98.8%
[27]	MLP Classifier with genetic search algorithm	98.1%
[28]	Random Subspace method with KNN classifier	97.2%
[29]	Gradient Boosting Machines (GBM)	97.5%
[30]	Deep learning with Convolutional neural Networks (CNN)	98.3%
[31]	XGBoost with feature selection	99.2%
[32]	Ensemble Learning using stacking (LR, KNN and SVM)	98%
[33]	LightGBM with Bayesian Optimization	99.0%
[34]	CatBoost With feature selection	98.2%
[35]	Extreme Learning Machines (ELM)	97.3%

**Table 2:** Comparison of Accuracy of fusion data model with other deep learning models.

Deep Learning Model	Accuracy
fusion Model (Proposed)	95.089%
GNN Model	92.958%
Tabular Model	90.987%
Baseline Model	85.249%

## Data Availability

To access the chronic Kidney disease dataset, interested researchers can visit the official website (https://archive.ics.uci.edu/ml/datasets/chronic_kidney_disease/ accessed on 1 July 2019). The CKD dataset is a publicly available strutured dataset that consists of 400 patients records.
